# Editorial: Termination of pro-inflammatory signaling and its dysregulation in autoimmune diseases

**DOI:** 10.3389/fimmu.2023.1174996

**Published:** 2023-03-10

**Authors:** René Huber, Daniela Novick, Guochang Hu

**Affiliations:** ^1^ Institute of Clinical Chemistry, Hannover Medical School, Hannover, Germany; ^2^ Department of Molecular Genetics, The Weizmann Institute of Science, Rehovot, Israel; ^3^ Department of Anesthesiology, University of Illinois College of Medicine, Chicago, IL, United States; ^4^ Department of Pharmacology and Regenerative Medicine, University of Illinois College of Medicine, Chicago, IL, United States

**Keywords:** resolution of inflammation, pro-inflammatory mediators, autoimmunity, termination of pro-inflammatory signaling, pharmacological modulation

Following acute inflammation, the events driving the immune response have to be ceased to enable the resolution of the inflammation and the restoration of homeostasis (1). Since the enduring exposure of cells to a pro-inflammatory milieu may lead to a prolonged, intense activation of intracellular signaling, removal of soluble mediators and accurate restriction of pro-inflammatory signaling in time are key features in this process. Dysregulation in this crucial step may result in unresolved inflammation (2) and the further development of chronic inflammatory disease or autoimmune disorder (1). The latter emerges in various systemic or organ-specific manifestations, such as rheumatoid arthritis (RA), psoriasis, inflammatory bowel disease (IBD), diabetes mellitus (DM), multiple sclerosis (MS), or systemic lupus erythematosus (SLE) (3). Despite the immense progress in elucidating the molecular basics of autoimmunity in the last decades, the landscape of disturbances contributing to the development and perpetuation of autoimmune disease has not been entirely explored. This article collection aimed at further identifying the molecular mechanisms involved in the various types of pro-inflammatory signal termination and the dysregulations that may contribute to the pathogenesis of autoimmune diseases.

In that context, the role of soluble mediators deserves special attention (4). In their review article, Xu et al. highlight the impact of the TNF superfamily member TNF-like cytokine 1A (TL1A) on innate and adaptive immunity and summarized its pro-inflammatory properties in autoimmune diseases. The authors discuss the role of TL1A in the activation of pro-inflammatory signaling, the production of cytokines, and the formation, differentiation, or recruitment of relevant cell types. Xu et al. also address the relevance of TL1A polymorphisms for the susceptibility to inflammatory diseases and the potential of anti-TL1A strategies. Equivalently, the review by Ihim et al. illustrates the novel role of the IL-1 family cytokine IL-18 in immunity, host defense, and the pathophysiology of autoimmunity. The authors comprehensively discuss the involvement of IL-18 in a broad range of diverse diseases with an inflammatory component. They also give special attention to IL-18 as a target of pharmacological intervention and the resulting clinical challenges.

In addition to physiological mediators, volatile organic compounds (VOC) have been shown to enhance inflammatory processes in animal models (5). Ogbodo et al. took up that topic by providing a survey of natural as well as artificial VOC from different sources as pro-inflammatory agents. Though the mechanism(s) of VOC-driven inflammation are not completely clear, the authors summarize the data available so far on VOC-induced oxidative stress, subsequent deregulation in pro-inflammatory gene expression (presumably mediated *via* redox-sensitive pathways), and the (over-) activation of immune cells resulting in deleterious immune responses. Another chapter is dedicated to the relevance of VOC-dependent changes in epigenetics as represented by alterations in DNA methylation patterns.

Given the detrimental inflammatory events in autoimmune diseases and the limitations of current therapeutic approaches (including non-responders and adverse effects), novel anti-inflammatory strategies are desirable (6). For the angiotensin II type 2 receptor (AT_2_R), for instance, a significant anti-inflammatory and tissue-protective capacity has been described (7). In their original research article, Sehnert et al. assess the therapeutic potential of the selective AT_2_R agonist compound 21 (C21) in a murine collagen-induced arthritis model and report promising immunomodulatory effects of C21. Both prophylactic and early therapeutic treatments with C21 significantly reduced the incidence and severity of arthritis. Further, C21 markedly decreased joint inflammation and cartilage destruction as well as the number of local IL-1β- and IL-17A-expressing immune cells, while increasing the number of regulatory T cells. Antibody production or neutrophil migration, however, were not affected. Another possible target was identified by Liu et al. using bioinformatics. Based on the gene expression omnibus database, the authors analyzed expression profiles from patients with psoriasis and Alzheimer’s disease (AD). Referring to Kyoto Encyclopedia of Genes and Genomes (KEGG) pathways, gene set enrichment analyses (GSEA) showed enrichment in metabolism- and biosynthesis-associated pathways for psoriasis and AD. Additional single sample GSEA indicated broad alterations in immunological processes, especially in psoriasis. Among the differentially expressed genes (DEG) present in both diseases, a set of common DEGs enriched in inflammatory and metabolic pathways and regulated by specific signature transcription factors (STF) were identified. Moreover, according to protein-ligand interaction analyses, these STFs may be targeted by TNF or IL-17A antagonists, which could open new pharmacological avenues. Subsequent analyses led to the prediction of ZNF384 as a potential master regulator of the STF network and thus, the inflammatory and metabolic events characterizing autoimmune diseases.

Finally, immunogenic cell death, as an essential pathophysiological step in autoimmunity, was revisited (Brieske et al.). Lai et al. review the contribution of ferroptosis, a distinct, iron-dependent form of cell death, to autoimmune disease. The research illustrates the characteristics of ferroptosis in comparison to other variants of cell death and - despite the still existing uncertainties about its exact molecular origin (8) - the current findings on critical mechanisms, which suggest that crucial disturbances in the prevention of lipid peroxidation are the decisive trigger of ferroptosis. Moreover, the authors extensively describe the opposing roles of ferroptosis in SLE, RA, and IBD, and discuss in detail the (complex) perspectives of modulating ferroptosis to suppress autoimmunity.

In summary, the manuscripts in this collection shed light on a selection of the multifaceted cellular and molecular alterations and disturbances leading to dysregulated inflammation at the onset of autoimmune disease. Occurrence and maintenance of these malfunctions may depend on a variety of different molecular entities (such as (bio-) chemical mediators, receptors, intracellular signaling molecules, and metabolic enzymes) and can impair multiple regulatory levels, including pro-inflammatory signal transduction, gene expression, and (energy) metabolism. The diversity of autoimmunity is reflected in the various cell types, tissues, and organs that can be affected and the multiple diseases in which autoimmunity may appear. The studies presented here also illustrate the promising but challenging avenues to anti-(auto-) inflammatory therapy and emphasize the necessity for novel and customized approaches to subdue autoimmunity.

## Author contributions

All authors listed have made a substantial, direct, and intellectual contribution to the work and approved it for publication.
